# The Pharmacopoeia of the United States of America

**Published:** 1894-01

**Authors:** 


THE PHARMACOPOEIA OF THE UNITED STATES OF AMERICA. Seventh Decennial Revision. 1890.

The committee in the preparation of this volume, have made at least one *radical* change, viz. the use of the *metric system* of weights and measures, to the entire exclusion of the old apothecaries system. This may cause some inconvenience for awhile, but we believe the change to be a wise one and hope that both physicians and pharmacists will give it their support.

The word *official* has been substituted for *officinal*. The list of articles dismissed from the Pharmacopeia is about equal to the new articles added, and no substance which cannot be produced except under a patent process or which is protected by proprietors' rights has been introduced.

